# Measuring Emotional Awareness in Patients With Schizophrenia and Schizoaffective Disorders

**DOI:** 10.3389/fpsyg.2021.725787

**Published:** 2021-11-11

**Authors:** Eva Maaßen, Marielle Büttner, Anna-Lena Bröcker, Frauke Stuke, Samuel Bayer, Jasmina Hadzibegovic, Sandra Anna Just, Gianna Bertram, Richard Rau, Dorothea von Haebler, Günter Lempa, Christiane Montag

**Affiliations:** ^1^Department of Psychiatry and Psychotherapy, Psychiatric University Clinic at St. Hedwig Hospital, Charité – Universitätsmedizin Berlin, Corporate Member of Freie Universität Berlin, Humboldt-Universität zu Berlin, and Berlin Institute of Health, Berlin, Germany; ^2^Psychoanalytic University Berlin, Berlin, Germany; ^3^Department of Psychology, University of Münster, Münster, Germany; ^4^Psychotherapy Practice, Munich, Germany

**Keywords:** schizophrenia, emotional awareness, affective mentalizing, theory of mind, levels of emotional awareness scale

## Abstract

The ability to mentalize (i.e., to form representations of mental states and processes of oneself and others) is often impaired in people with schizophrenia spectrum disorders. Emotional awareness (EA) represents one aspect of affective mentalizing and can be assessed with the Levels of Emotional Awareness Scale (LEAS), but findings regarding individuals with schizophrenia spectrum disorders are inconsistent. The present study aimed at examining the usability and convergent validity of the LEAS in a sample of *N* = 130 stabilized outpatients with schizophrenia or schizoaffective disorders. An adequacy rating was added to the conventional LEAS rating to account for distortions of content due to, for example, delusional thinking. Scores of the patient group were compared with those of a matched healthy control sample. Correlation with symptom clusters, a self-report measure of EA, a measure of synthetic metacognition (MAS-A-G), and an expert rating capturing EA from the psychodynamic perspective of psychic structure (OPD-LSIA) were examined. Regarding self-related emotional awareness, patients did not score lower than controls neither in terms of conventional LEAS nor in terms of adequacy. Regarding other-related emotional awareness, however, patients showed a reduced level of adequacy compared to controls whereas no such difference was found for conventional LEAS scores. Higher conventional LEAS scores were associated with fewer negative symptoms, and higher structural integration of self-perceptions measured by the OPD-LSIA. Higher adequacy of responses correlated with fewer symptoms of disorganization as well as excitement, higher scores of self-reflection on the MAS-A-G as well as self- and object-perception and internal and external communication as measured by the subscales of the OPD-LSIA. Findings suggest that the LEAS might not be sensitive enough to detect differences between mildly symptomatic patients with schizophrenia or schizoaffective disorders and healthy controls. However, LEAS ratings are still suitable to track intraindividual changes in EA over time. Observing the adequacy of patients’ responses when using the LEAS may be a promising way to increase diagnostical utility and to identify patterns of formal and content-related alterations of mentalizing in this patient group. Methodological indications for future studies are discussed.

## Introduction

Mentalizing describes the capacity to form representations of mental processes and to reflect on one’s own and others’ inner states (Fonagy and Bateman, 2016). Deficits in mentalizing abilities have been shown to be prominent in schizophrenia spectrum disorders (SSD) and are associated with aversive interpersonal experiences (Hasson-Ohayon et al., 2015) and functional impairment (Fett et al., 2011). Specific impairments that have been found in SSD patients include deficits in basic aspects of emotion processing (Tremeau, 2006) as well as in various aspects of theory of mind (ToM; Frith, 2004; Sprong et al., 2007) and metacognition (Lysaker et al., 2011a). Studies reporting impairments in the affective rather than cognitive components of mentalizing such as empathy (Derntl et al., 2009; Montag et al., 2020) are less common and there is only a limited spectrum of performance-based approaches to measure them (Bonfils et al., 2016). Yet, the improvement of affective mentalizing abilities is receiving increasing attention in psychotherapies for patients with schizophrenia (Liotti and Gilbert, 2011; Lempa et al., 2013; Montag, 2015; Weijers et al., 2020), and valid and reliable instruments are needed in order to investigate their effectiveness.

One important indicator for affective mentalizing abilities is emotional awareness (EA). Being aware of an affective state—rather than focusing on non-emotional aspects of an interpersonal situation—is necessary to use intentional regulation strategies on an intrapersonal and interpersonal level (Barrett et al., 2001; Decety and Jackson, 2004) or as Lane (1991, p. 4) puts it: “How can we resolve our anger if we’re not aware that we are angry?” However, while Kimhy et al. (2012) in their conception of EA emphasize the ability to be attentive to one’s own emotions, to distinguish and to use discrete verbal labels to describe them, Lane et al. (2015) define the term more broadly: EA enables a person to imagine an affective state in oneself or another and thus to (vicariously) experience it, to form mental representations of emotional states and to draw inferences on this basis—thus being closer to the concept of affective mentalizing.

To capture EA, Lane et al. (1990) developed the Levels of Emotional Awareness Scale (LEAS) based on Piaget’s stages of cognitive development. The need for a measure such as the LEAS arose in the context of alexithymia research, but higher LEAS scores have also been shown to be an indicator of affective mentalizing skills in patients with somatoform disorders (Subic-Wrana et al., 2010, 2011; Stonnington et al., 2013; Lane et al., 2015). LEAS scores were also found to be lower in cocaine addiction when it was associated with impaired insight (Moeller et al., 2010). The LEAS uses scenarios of social situations to evoke participants’ emotions by simulation and assess both their own emotional reactions and their inferences about the emotions of interaction partners presented in the scenario in an open response format. For example, one scenario asks participants to imagine repairing a piece of furniture for their neighbor and accidentally hitting their finger with a hammer (Scenario 1). They are then asked “How would you feel in this scenario?” (*Self* score) and “How would the other person feel in this scenario?” (*Other* score). Later, the complexity of the emotional terms is rated with higher scores indicating more differentiated EA (e.g., the ability to name global vs. ambivalent feelings). The objectivity of the rating is ensured by providing raters with a standardized glossary listing scores of words used in previous answers. Compared to other performance-based measures of affective mentalizing, the LEAS has the advantage of being highly efficient, easy to administer, objective, and reliable (Lane et al., 1990; Subic-Wrana et al., 2011).

If the LEAS is as suitable to measure EA as an indicator of affective mentalizing in people with SSD as it is in people with psychosomatic disorders (Subic-Wrana et al., 2010; Lane et al., 2015), it should be able to differentiate between patients and healthy control subjects. However, studies comparing LEAS scores of individuals with SSD to healthy controls have yielded inconsistent findings. Baslet et al. (2009) found lower LEAS scores in individuals with schizophrenia or schizoaffective disorders for the *Other* subscale and complex scenarios only, but not for the *Total* or any other subscales. Henry et al. (2010) reported lower LEAS scores for the *Self* subscale but no difference for the *Other* or the *Total* scale in individuals with schizophrenia or schizoaffective disorders. Other studies found lower LEAS scores for both subscales in individuals diagnosed with schizophrenia compared to healthy controls (Harrison et al., 2007; Jáni et al., 2021). Li et al. (2019) found no difference in any of the scales in individuals with schizotypy compared to healthy controls.

One possible explanation for these inconsistent findings might be the rather small sample sizes in previous studies. However, another reason could be the lack of consideration of the adequacy of the named emotions with respect to the provided context, as the nature of the inferred emotion could be altered in a disease-specific manner: Frewen et al. (2008) reported reduced adaptability of stated emotions in people with posttraumatic stress disorder (PTSD): Some patients exhibited disorder-specific distortions of their emotional experience, like feeling shame and aversion facing a well-intentioned back massage (LEAS scenario 3). The authors therefore established a rating to assess both formal and content-related characteristics of the responses. Similarly, in patients with SSD, formally high LEAS scores may be contextually adequate or not—a patient may expect concern, ignorance or even glee from his neighbor, who witnessed him hitting his thumb with a hammer (LEAS scenario 1). Frith (1992, 2004) was the first to distinguish between subgroups of patients with schizophrenia, who either showed a difficulty to conceptualize representational mental states at all, or had an intact ability to represent mental states, but inaccurately ascribed internal states to other people. This might imply concretist or overly simplistic mental state attributions (“undermentalizing”) in the first, and hyper-developed, excessive inferences about others’ mental states in the second condition. In line with this, a hyper theory of mind (Abu-Akel and Bailey, 2000) or “overmentalizing” (Frith, 2004) was found to be associated with positive psychotic, i.e., delusional, while undermentalizing was related to negative symptoms (Montag et al., 2011; Fretland et al., 2015). This suggest that high LEAS scores might not exclusively reflect the ability to engage in affective mentalizing but might also be produced by a tendency to overmentalize. A tendency to undermentalize in the context of predominant negative symptoms could result in low LEAS scores combined with low, moderate, or even normal adequacy. For example, in scenario 1, the neighbor could be attributed an action tendency rather than an emotion (e.g., “The neighbor would say *Ouch*”), which would result in a low LEAS score, but this would still be an empathic and socially appropriate (though undermentalized) attribution. Thus, to reveal whether symptoms are related to affective mentalizing, it might be necessary to consider both the complexity and the adequacy of expressed emotions as well as different symptom clusters.

Beyond differentiating between patients and controls, another feature that may demonstrate the suitability of the LEAS as a measure of affective mentalizing in individuals with SSD is criterion-related validity. To this end, we examined the overlap of LEAS scores with three measures. The Mentalization Questionnaire (MZQ; Hausberg et al., 2012) served as a non-performance-based, self-reported measure of mentalizing capability. The Metacognition Assessment Scale—Abbreviated (MAS-A; Lysaker et al., 2005) captures synthetic metacognition (i.e., the ability to “think about thinking” Lysaker et al., 2011b) as a different, but overlapping concept to EA, e.g., identifying different emotional states in oneself and others and perspective taking are important for both concepts. The *Levels of Structural Integration Axis* of the Operationalized Psychodynamic Diagnostics/OPD-2 (OPD-LSIA; OPD Task Force, 2014) reflects the availability of mental functions like perception, communication, regulation, and attachment. Aspects of EA are found in the description of the dimensions of self- and object perception as well as communication to the internal and external world (Cierpka et al., 2007).

The goals of the present study were fourfold. First, considering the inconsistency of prior findings, we sought to investigate potential impairments in EA related to SSD more comprehensively by comparing LEAS scores from a large sample of patients diagnosed with schizophrenia or schizoaffective disorders to a matched sample of healthy controls, and lower LEAS scores were expected in patients compared to controls. We conceived it as a potential shortcoming that the LEAS conventionally involves only an evaluation of the formal complexity, but not of the adequacy of responses. Inspired by Frewen et al. (2008), in a second step, we therefore collected additional expert ratings on the adequacy of responses and compared it between patients and controls. We assumed that patients would show a tendency toward lower adequacy scores compared to healthy individuals.

Third, we examined whether symptomatology was related to EA within the group of patients. One could argue that negative associations should be expected since intact mentalizing was robustly associated with fewer symptoms (Fretland et al., 2015). However, based on considerations of over- or undermentalizing, associations with symptom clusters might point in different directions (i.e., high EA in the context of positive, low EA in the context of negative symptomatology; Abu-Akel and Bailey, 2000; Frith, 2004). We therefore expected conventional LEAS scores to show a negative correlation with negative, but not with positive symptoms. We further expected adequacy scores to show negative correlation with positive, but not with negative symptoms. For the other symptom clusters, correlations with LEAS and adequacy scores were examined on an exploratory basis.

Moreover, we sought to explore the LEAS criterion-related validity more deeply. We expected moderate convergence with the MZQ as a non-performance-based measure of EA and with the subscales of the MAS-A (Lysaker et al., 2005) tapping also the non-affective aspects of mentalization for oneself and others. EA-related aspects of psychic structure captured with the OPD-LSIA (OPD Task Force, 2014) were expected to positively correlate with the LEAS. Finally, to test whether adequacy rating could improve the criterion-related validity of the LEAS, we examined the same correlations for this alternative scoring of the LEAS.

## Materials and Methods

The present work is based on data from the baseline survey of the ongoing study *Modified Psychodynamic Psychotherapy for Patients with Schizophrenia—A Randomized-Controlled Efficacy Study* (MPP-S; ClinicalTrials.gov-ID: NCT02576613). The study was approved by the local ethics committee, all patients gave fully informed consent.

### Participants

Participants were 130 outpatients recruited from the Department of Psychiatry, Charité Universitätsmedizin Berlin, and International Psychoanalytic University, Berlin. All participants met criteria for schizophrenia (74.6%) or schizoaffective disorders (25.4%) according to DSM-IV-TR (Saß et al., 2003). The diagnostic assessment was carried out by an experienced psychiatrist using a structured interview (SCID-I; First, 2015), complemented by any available information like hospital discharge letters. The sample consisted of 74 male (56.9%) and 56 female (43.1%) patients and age ranged from 19 to 63 years. The mean duration of illness was 13.19 years (± 9.79) and the mean value of the *Global Assessment of Functioning* (GAF) was 58.57 (± 12.71). Medication protocols were as follows: unmedicated: *n* = 12; atypical neuroleptic: *n* = 106; conventional neuroleptic: *n* = 3; combination atypical + conventional neuroleptic: *n* = 7; additional mood stabilizer: *n* = 9; additional antidepressant: *n* = 28. A healthy control sample that was matched for age, education, and gender was kindly provided by Richard D. Lane (*n* = 129). The subjects in the control sample were a subgroup from a larger sample (*N* = 380) that Lane et al. (1996) collected in the United States to obtain a LEAS norm sample stratified by age, sex, and socio-economic status (see Lane et al., 1996 for more detailed information). *N* = 94 matches could be based on exact matching for sex, age within a range of 5 years and education degree (seven categories: *none; Grades 1*–*9; Grades 10*–*11; High school graduate; Some college; College graduate; more than college degree*). *N* = 35 matches could be based on sex and age, but controls had significantly higher levels of education than patients (Table 1).

**TABLE 1:** Demographic data from patient and control group.

**Variables**	**Patients**	**Controls**	**Statistics**	
	***N* = 130**	***N* = 129**	***t***	***p***
Age^1^	38 ± 11.3	38.1 ± 11.7	−0.103	0.918
Gender (m/f)^2^	74/56	74/55	χ^2^ = 0.005	0.934
Education degree^1^	4.5 ± 1.5	5.3 ± 1.2	−5.084	0.000

*Between group comparison: ^1^*t*-test for independent samples (two-tailed): mean ± standard deviation; ^2^χ^2^-test.*

*Educational degree categories: (0), none; (1), Grades 1–9; (2), Grades 10–11; (3), High school graduate; (4), Some college; (5), College graduate; (6), more than college degree.*

### Levels of Emotional Awareness Scale

Originally, the LEAS consists of 20 scenarios (Lane and Schwartz, 1987). In the present study, two statistically parallel versions, A and B, of 10 items each (Subic-Wrana et al., 2011) of the German version translated and validated by Subic-Wrana et al. (2001) were used. Half of the sample completed version A, the other half completed version B. This was done to avoid learning effects in context of the longitudinal study. The LEAS was administered orally by trained interviewers and audio-recorded. Roberton et al. (2020) found no difference between the LEAS scores in dependence on the administration mode (oral vs. written). The interviewers were instructed not to ask follow-up questions (e.g., “But how would you feel?” in case of reported thoughts) or reinforce patients’ responses in any manner. The patients’ responses were hand-scored by a trained rater using the provided German glossary (Subic-Wrana et al., 2001) and subsequently reviewed by another trained rater. The glossary provides a respective score for possible answer words from (0) *no awareness*, (1) *physical sensation*, (2) *action tendencies implying emotions and valenced non-specific emotions* (e.g., *good, bad*) to (3) *specific or complex emotions* (e.g., *happy)* words used in previous answers. The score (4) is obtained if at least two distinguishable emotions scored with (3) are mentioned in the answer. If a word is not found in the glossary, raters are advised to look for a comparable word. The *Total* score is the higher of the *Self* and the *Other* scores. If *Self* and *Other* score are at level (4), a *Total* score of (5) is given if the scored words for *Self* and *Other* are not the same. The responses of the English-language control sample that were administered in a written mode were translated into German and then evaluated using the same glossary as for the patients. Raters were blinded to LEAS scores from the original American rating. To check whether there had been any bias due to translation, the interrater reliability between the LEAS values of the German rating and the American rating was calculated. The evaluation followed the scoring described above.

#### Adequacy Rating

For the adequacy rating, all given answers (patients and controls) were extracted and presented in German to seven raters. Following the scoring method of LEAS, individual words were scored for adequacy (*0* = *inadequate, 0.5* = *somewhat adequate, 1* = *adequate*), but with reference to one specific scenario. Thus, the same word could receive different adequacy scores in different scenarios. The operationalization for the adequacy rating was as follows: *adequate/appropriate* = plausible in the common sense meaning, appropriate to the social situation depicted—*somewhat adequate* = deviating from common sense, but adequate/still somehow understandable, when taking in consideration the sense of patients personal meaning—*inadequate* = no plausible sense at all, inappropriate in context of the social situation depicted. All raters had at least 2 years of professional experience in the therapeutic setting. Raters were blind to the origin of the responses (patients or healthy controls) as well as to the conventional LEAS score given for the response. Two raters were excluded due to low variance, a high rate of missing values and negative correlation with the other raters. Subsequently, mean scores across the five remaining raters were calculated regarding the adequacy of an answer related to the specific scenario. Following the conventional LEAS rating, the mean values were used to determine a *Self*, an *Other*, and a *Total* score for each subject. For the *Self* and the *Other* scores, responses of a subject to every scenario were evaluated for adequacy and the mean score for the responses was calculated. The *Total* score results from the sum of the *Self* and the *Other* score. We will refer to the scores obtained as described in Lane et al. (1990) as *conventional LEAS* scores and to the ones based on adequacy ratings as *adequacy* scores. Adequacy scores correlated only moderately with conventional LEAS scores (*Total*: *r* = 0.215, *p* = 0.001; *Self*: *r* = 0.327, *p* = 0.000, *Other*: *r* = 0.274, *p* < 0.000) indicating the overlap is positive and statistically significant but explains only a small amount of variance and thus the two measures are partially independent.

### The Positive and Negative Syndrome Scale

The Positive and negative syndrome scale (PANSS; Kay et al., 1987) is an expert rating based on 30 items. The presence of positive, negative, and general symptoms of schizophrenia are rated on a 7-point Likert scale, ranging from *1* = *absent* to *7* = *extreme*. In the present study, the five factor model revised by van der Gaag et al. (2006) was used instead of the initial three factor model. It distinguishes positive symptoms (i.e., delusions, grandiosity), negative symptoms (i.e., blunted affect, social withdrawal), disorganization (i.e., stereotyped thinking, disorientation), excitement (i.e., hostility, poor impulse control) and emotional distress (i.e., depression, anxiety, tension). Each of the five factors consists of 8 to 10 items. Higher scores indicate a higher symptom load. Ratings reflected the consensus of two clinical psychologists from a pool of trained raters based on a larger interview (see section “Mentalization Questionnaire, German version of the Metacognition Assessment Scale and Operationalized Psychodynamic Diagnostics-2”).

### Validation Tasks

#### Mentalization Questionnaire, German version of the Metacognition Assessment Scale and Operationalized Psychodynamic Diagnostics-2

The *mentalization questionnaire* (MZQ; Hausberg et al., 2012) is a questionnaire based on self-report about the ability to perceive emotions in oneself and others. It consists of 15 items answered on a 5-point Likert scale. The MZQ consists of four subscales: refusing self-reflection (MZQ 1), emotional awareness (MZQ 2), psychic equivalence mode (MZQ 3) and regulation of affect (MZQ 4) as well as a total score (MZQ total) across all 15 items. Higher scores indicate lower mentalizing abilities.

The *Metacognition Assessment Scale—Abbreviated* (MAS-A; Lysaker et al., 2005) is an expert rating on synthetic metacognitive skills. It consists of four subscales: self-reflectivity (SR; nine items), understanding the other’s mind (OR; seven items), decentration (the ability to abstract from one’s own perspective; four items) and mastery (capacity to solve psychological and interpersonal problems using metacognitive knowledge; nine items). Higher scores indicate higher metacognitive abilities. We used a German version validated by our research group (MAS-A-G; Bröcker et al., 2017).

The *Operationalized Psychodynamic Diagnostic-2* (OPD-2; OPD Task Force, 2014) assesses individual differences on five different axes: (I) experience of illness, (II) interpersonal relations, (III) conflict, (IV) psychic structure, and (IV) psychological and psychosomatic disorders. In the context of psychodynamic theories, individual problems become particularly understandable against the background of a repetitive relational experience (axis II), inner conflicts (axis II) and the availability of psychological functions (axis IV). The levels of structural integration axis (OPD-LSIA, Axis IV) was of particular interest for the present work as it captures aspects of EA on four of its eight subscales, namely *(reflexive) self and other perception* (mapped by, e.g., self-reflection, differentiation of affects, and holistic and realistic perception of others) and *communication to the internal and external world* (mapped by, e.g., ability to experience and communicate affects or empathy). The psychic structure is assessed on a scale from *(1) well integrated* to *(4) disintegrated*, thus lower scores indicating higher level of integration.

The MAS-A-G and the OPD-LSIA rating were based on a half-structured interview that lasted between 60 and 90 min. The interview was conducted by two investigators from a pool of clinical psychologists trained in interview and rating principles. They asked questions about levels of social functioning and symptomatology but also encouraged the patients to talk freely about significant relationships or life events that were important to them in their past (see Bröcker et al., 2017; Stuke et al., 2020 for a detailed description). MAS-A-G and OPD-LSIA ratings are based on consensus rating of the respective investigators who conducted the interview.

#### Control Variables

As several studies indicate an influence of general cognitive functions on mentalizing abilities (Greig et al., 2004; Brüne, 2005) we assessed two measures of cognitive ability as control variables. Specifically, we used a 40-item, multiple-choice, vocabulary test (Wortschatztest, WST; Schmidt and Metzler, 1992) to assess the “premorbid” verbal intelligence level and the Auditory Verbal Learning Test (AVLT; Heubrock, 1992) to assess verbal memory, verbal learning, and executive function. Following convention, the mean score of the first five trials was used for analysis [AVLT^(1–5)^]. Descriptive statistics are displayed in Table 2.

**TABLE 2:** Characteristics of illness, validation tasks, and control variables in patient sample.

	***M***	**SD**
PANSS negative	16.46	6.68
PANSS positive	14.76	6.0
PANSS disorganization	17.79	5.47
PANSS excitement	13.76	3.41
PANSS emotional distress	18.54	5.95
MZQ 1	8.99	4.11
MZQ 2	13.88	3.89
MZQ 3	10.78	3.8
MZQ 4	10.58	2.73
MZQ total score	37.2	11.58
MAS-A-G SR	6.46	1.73
MAS-A-G OR	4.21	1.23
MAS-A-G D	1.82	0.82
MAS-A-G M	5.54	1.65
OPD-2 Self-perception	2.53	0.62
OPD-2 Object Perception	2.98	0.6
OPD-2 Internal communication	2.71	0.59
OPD-2 Communication with the external world	2.60	0.59
Verbal Intelligence (WST)	105.74	11.86
AVLT^(1–5)^	9.07	2.48

*M, mean; SD, standard deviation; PANSS, Positive and Negative Syndrome Scale; WST, the premorbid intelligence level; MZQ: MZQ 1, refusing self-reflection; MZQ 2, emotional awareness; MZQ 3, psychic equivalence mode; MZQ 4, regulation of affect; MZQ total; MAS-A-G, German version of the Metacognitive Assessment Scale- SR, self-referential; OR, object-referential; D, decentration; M, mastery; OPD-2: LSIA-OPD subscales for: self-perception, object perception, internal communication, and communication to the external world; AVLT^(1–5)^ = auditory verbal learning test: mean score of the first five presentations.*

### Statistical Analyses

All statistical analyses were performed in SPPS 27.0 (Ibm Corp, 2020). Inter-rater reliability was estimated as the intraclass-correlation (ICC) using an absolute-agreement, 2-way random-effects model (Koo and Li, 2016). Mann–Whitney-Tests was used to compare the group means of patients vs. controls and Spearman correlation coefficient was used to assess the linear relationship between LEAS scores and the validation variables. Given our general expectation that higher LEAS scores would be associated with better psychological adjustment, we evaluated statistical significance against a one-sided alpha-level of 5% in the majority of cases (except for three symptom clusters of the PANSS which were examined two-sided on an exploratory basis). Given the large number of tests, we also report which tests pass a Bonferroni-corrected significance criterion. Finally, we note that the amount of available data varied slightly between analyses due to selectively missing data and outlier exclusions. Details on missing data and exclusions are presented in Supplementary Appendix 1.

## Results

### Preliminary Analyses

First, we assessed the interrater reliability of our LEAS data. ICC for the control group was computed to ensure sufficient interrater reliability and to support the validation of the raters’ manual. This was particularly important to rule out biases due to translation errors from English to German. Analysis revealed an ICC of 0.89 for the *Total* score, 0.91 for the *Self* score and 0.92 for the *Other* score between Lane’s and our rating for the control group. Then, ICC between raters (*k* = 5) for adequacy of LEAS answers for patient and control group was examined. ICCs for the different scenarios ranged from 0.49 to 0.86 (*Self*) and from 0.70 to 0.94 (*Other*) (Supplementary Appendix 2). Poor interrater reliability (ICC < 0.50) pertained to the *Self* score of one scenario, but the remaining ICC may be classified as moderate to excellent (Koo and Li, 2016). Overall, raters’ average adequacy scores were quite high [*M*(*Self*) = 0.84 (*SD* = 0.06); *M*(*Other*) = 0.75 (*SD* = 0.08)] which may suggest a ceiling effect. In our sample, patients with schizophrenia did not differ significantly from patients with schizoaffective disorders in their mean conventional LEAS or adequacy scores on any subscale which is why both subgroups were examined together and referred to as patient sample.

### Group Differences in Levels of Emotional Awareness Scale Rating

Levels of Emotional Awareness Scale results are displayed in Table 3. Mann–Whitney-Tests were conducted to assess differences in conventional LEAS scores between patient and control group. Firstly, the analysis was calculated with both versions combined. Then, differences between groups were assessed separately for version A and version B, to confirm content-related overlap. Overall, conventional LEAS scores did not differ between the groups for neither of the three scales.

**TABLE 3:** Comparison of conventional LEAS and adequacy mean scores of patients and control group.

**Scoring**	**Scale**	**Patients**		**Controls**		**Group difference**	
		***M***	**SD**	***M***	**SD**	***z (df)***	***p***
LEAS rating	Total	30.93	4.41	30.73	5.11	−0.486 (246)	0.314
	Self	26.59	4.86	27.07	5.79	−0.873 (246)	0.191
	Other	24.84	5.30	24.36	5.36	−0.763 (246)	0.223
Adequacy rating	Total	16.77	1.64	17.08	1.51	−1.593 (244)	0.056
	Self	8.86	0.90	8.86	0.66	−1.267 (244)	0.102
	Other	7.95	0.96	8.22	1.11	−2.513 (244)	0.006**

*M, Mean; SD, standard deviation; df, degree of freedom.*

Mann–Whitney-Test (one-sided), **p < 0.01.

#### Group Differences in Adequacy

Analysis was calculated with both versions combined. Response adequacy differed between groups for *Other* but neither for *Total*, nor for *Self* (see Table 3). The patient group showed higher inadequacy in their answers regarding *Other*. Effect size according to Cohen was *r* = 0.16 for *Other* scale indicating a small effect.

### Conventional Levels of Emotional Awareness Scale Rating and Adequacy Rating Correlations

Correlations for LEAS *Total*, *Self*, *Other* and PANSS five factors were computed to analyze associations between clinical symptoms and LEA as well as adequacy (see Table 4). The results show that higher scores in conventional LEAS and adequacy tended to be associated with fewer symptoms. Conventional LEAS scores tended to rather inversely correlate with negative, but not positive symptoms. Adequacy scores tended to rather inversely correlate with positive but not negative symptoms. Highest coefficients were found for adequacy and symptoms of disorganization and excitement. Partial correlations *controlling f*or verbal IQ and AVLT^(1–5)^ were computed to assess associations between the additional tasks and conventional LEAS and adequacy ratings. Only the MZQ scale “psychic equivalence mode” correlated with conventional LEAS *Self* and *Other* to a small degree. Correlations between MAS-A-G scales and conventional as well as adequacy rating were found, indicating that higher ratings on metacognitive abilities are associated with higher conventional LEAS and higher adequacy scores. In addition, for both ratings correlations with the four scales of interest of the OPD-LSIA of the were found. Conventional LEAS *Total, Self* and *Other* inversely correlated with self-perception to a small degree, demonstrating that higher scores on the conventional LEAS were associated with better structural integration regarding self-perception. Adequacy ratings showed correlations with all OPD-LSIA scales such that higher adequacy values were associated with higher structural integration.

**TABLE 4:** Results of the correlation analysis (Spearman correlation coefficients).

	**LEAS scores**			**Adequacy scores**		
	**Total**	**Self**	**Other**	**Total**	**Self**	**Other**
		
**Variables**	***r***			***r***		
PANSS positive	–0.06	–0.05	–0.02	–0.18	–0.20	–0.10
PANSS negative	–0.22	–0.10	–0.14	–0.09	–0.08	–0.07
PANSS disorganization^a^	–0.16	–0.12	–0.14	–0.22	–0.25	0.15
PANSS excitement^a^	–0.06	–0.08	–0.04	–0.21	–0.21	–0.18
PANSS emotional distress^a^	–0.14	–0.10	–0.11	–0.03	–0.13	–0.01
MZQ 1	–0.04	–0.03	–0.06	0.14	–0.15	–0.13
MZQ 2	–0.09	–0.13	–0.00	–0.02	–0.05	0.09
MZQ 3	–0.15	–0.18	–0.18	–0.00	–0.00	–0.04
MZQ 4	–0.03	0.04	0.09	0.10	0.10	0.16
MZQ total score	–0.03	–0.02	–0.10	–0.08	0.06	–0.12
MAS-A-G SR	0.11	0.08	0.04	0.24	0.19	0.17
MAS-A-G OR	0.19	0.14	0.19	0.13	0.15	0.03
MAS-A-G D	0.13	0.07	0.16	0.15	0.11	0.12
MAS-A-G M	0.13	0.05	0.12	0.13	0.07	0.11
OPD-2 Self-perception	–0.23	–0.24	–0.16	–0.25	–0.19	–0.18
OPD-2 Object Perception	–0.13	–0.10	–0.11	–0.21	–0.15	–0.17
OPD-2 Internal communication	–0.12	–0.05	–0.11	–0.17	–0.14	–0.15
OPD-2 Communication with the external world	–0.17	–0.14	–0.15	–0.18	–0.14	–0.12

*PANSS, Positive and Negative Syndrome Scale; MZQ: MZQ 1, refusing self-reflection, MZQ 2, emotional awareness, MZQ 3, psychic equivalence mode, MZQ 4, regulation of affect, MZQ total; MAS-A-G, German version of the Metacognitive Assessment Scale- Abbreviated: SR, self-referential, OR, object-referential, D, decentration, M, mastery; OPD-2, LSIA-OPD subscales.*

*^*a*^Spearman correlation two-sided.*

*Corrected *p*-value (Bonferroni) for MAS-A-G, OPD-structure = *p* < 0.002; corrected *p*-value (Bonferroni) for PANSS = *p* < 0.003; corrected *p*-value (Bonferroni) for MZQ = *p* < 0.003.*

*No coefficient maintained significance after alpha-level correction.*

## Discussion

Previous findings regarding emotional awareness (EA) measured with the Levels of Emotional Awareness Scale (LEAS) are inconsistent with regard to individuals with schizophrenia spectrum disorder (SSD). The aim of the present study was to examine the instrument in a larger sample of patients diagnosed with schizophrenia and schizoaffective disorders relative to healthy controls, particularly focusing on context-related adequacy of responses and possible associations with symptoms. Moreover, indications of convergent validity were explored *via* correlational analysis.

Contrary to our expectations, conventional LEAS did not significantly differ between patients and healthy controls, neither on any of the subscales, nor when separating for version A and B. These findings are not consistent with a previous study by Jáni et al. (2021) who found lower scores for patients with schizophrenia on both subscales for the LEAS version A. Other authors found significantly lower LEAS scores for patients diagnosed with schizophrenia or schizoaffective disorders but only for either the *Other* (Baslet et al., 2009) or the *Self* subscale (Henry et al., 2010). Our finding might suggest that stabilized outpatients diagnosed with schizophrenia or schizoaffective disorders do not differ from healthy individuals in their formal EA, i.e., they can imagine, differentiate, and communicate different, even ambiguous, emotions and thus formally show as mature levels of EA as healthy persons. This is in line with the idea that patients with paranoid schizophrenia are capable of representing mental states but might draw faulty conclusions (Frith, 2004). Whether these are adequate in the context of the social situation in which they are experienced is not considered in the conventional LEAS scores which is why we additionally considered the adequacy of responses.

The adequacy of responses was lower in patients compared to healthy controls when responses referred to the emotions attributed to the other. This is in line with studies that reported emotional perspective-taking or affective ToM to be deficient in individuals with schizophrenia (Lee et al., 2011; Bonfils et al., 2016). Contrary to our expectation though, no difference in adequacy between patients and controls was found for self-related emotions. This may indicate that perspective-taking is the most challenging aspect of mentalizing and relates to the difficulties these patients have in social interaction. However, the result is still surprising, as LEAS scenarios are social situations in which attributions to the other person can also have effects on one’s own feelings. For example, in LEAS scenario 8, the question is how oneself and one’s boss would feel if the boss told one that the work done was deficient. A person who inadequately attributes sadistic joy to a criticizing boss might feel differently, and possibly more inappropriate regarding the specific social situation, than someone who attributes less extreme feelings to the boss. We therefore formulated the hypothesis globally across *Self* and *Other*, which was only partially confirmed.

The issue might be explained against the background of the test for interrater reliability (IRR) of the adequacy rating. IRR of adequacy ratings between five included raters were higher for other- than for self-related emotions such that raters were less in agreement about which self-referential responses should be evaluated as an adequate reaction in a certain situation. This might be due to the fact that the raters placed themselves in the situation to evaluate adequacy and thus judged the *Self* condition more subjectively than the *Other* condition. The idea behind selecting raters with a psychotherapeutic profession was to make such subjectivity bias less likely. However, psychotherapists may refrain from judging individual feelings on a normative basis, but rather accept them as valid and unique and support patients in understanding their origin. This reasoning is also backed up by the finding that self–related adequacy was consistently rated higher than other-related adequacy. For this reason, subtle differences between patient and control groups in the *Self c*ondition might have remained undetected.

Concerning the correlation analyses discussed hereafter, it must be stressed at the outset that correlation coefficients were small, many below *r* = 0.2, and not maintaining significance after Bonferroni correction. We would still like to discuss possible explanations to inspire subsequent work. Results might indicate that low formal levels of EA measured by conventional LEAS scores are more likely to parallel deficits in affective mental state representation as they occur with dominant negative symptomatology, whereas content-related adequacy in mental state reasoning might be subject to distortions related to positive symptoms (Frith, 2004). The 5-factor solution of the PANSS (van der Gaag et al., 2006) allows for the differentiation of the positive symptom cluster from disorganization, including symptoms such as stereotyped thinking and difficulty in abstraction, and excitement, capturing e.g., hostility and poor impulse control. Our findings at least do not contradict the idea that mild to moderate levels of positive symptoms, disorganization and excitement may impact content-related mental state inferencing, rather than the level of EA. Analyses might be more meaningful in samples with a higher symptom load, as the range of PANSS scores was rather restricted in our stabilized outpatients with moderate symptom severity (see Table 2 for PANSS mean values in the patient sample) and mild to moderate expression of delusions or hallucinations. Moreover, negative symptom might be further differentiated, e.g., in amotivation versus diminished expression as proposed by Fervaha et al. (2014) or expressive and experiential deficits as proposed by Jang et al. (2016).

Even though the conventional LEAS score did not detect impairments in EA among patients when compared to healthy controls, the measure might still be suitable to capture modifications in EA through psychotherapy interventions on an intrapersonal level. For instance, the LEAS was successfully used to track treatment-related changes in a pilot RCT of art psychotherapy for patients with schizophrenia (Montag et al., 2014). To strengthen this assumption, correlations with validated instruments for EA and overlapping constructs were examined.

Correlations with the self-report of the MZQ were low and not significant for both, the conventional LEAS as well as the adequacy rating. This is in line with previous studies indicating low correlation between self-ratings and objective or performance-based measures in patients with schizophrenia (Derntl et al., 2012; Smith et al., 2014).

Although both the MAS-A-G and the LEAS capture the increasing complexity of representations of self and other, associations were only small, and significance not maintained after alpha-level correction. The fact that the MAS-A-G assessment is based on an interview to elicit patients’ free narratives of personally relevant biographic and relationship episodes, whereas the LEAS captures EA using standardized scenarios of relatively low emotional intensity, might explain the little overlap between the instruments.

The third measure of interest was the OPD-LSIA that captures aspects of EA from a psychodynamic perspective. Higher scores on both conventional LEAS and adequacy ratings were associated with better structural integration. Highest correlations were found with the structural dimension of self-perception, both for conventional LEAS scores and adequacy sores. Thus, LEAS scores, and especially the adequacy scores, are associated with self-reflection, affect differentiation and a basic sense of self-identity (as part of the self-perception dimension, OPD-2; OPD Task Force, 2014). EA and its adequacy were also associated with the dimensions communication to the external and internal world. The ability to communicate to the external world (including making contact, affect communication, and empathy; OPD-2; OPD Task Force, 2014) is important for interpersonal affect regulation. Thus, the results might support the thesis that (adequate) EA is important to regulate emotions on an inter- and intrapersonal level and thus describes an important structural skill (Kimhy et al., 2012).

In summary, results point to inadequacies in other-related mentalizing in the patient group, while formal levels of EA did not differ significantly from healthy controls. This is in line with conceptions of intact formal mental state representation, but partially wrong conclusions in terms of content, albeit not necessarily in paranoid patients as proposed by Frith (2004). Deficits in stabilized outpatients seem to be much smaller than expected and confined to other-, not the self-related, aspects of mentalizing. Results from the correlation analyses indicate a partially convergent validity of the conventional LEAS and other measures capturing EA (OPD-LSIA) or related abilities (MAS-A-G). The adequacy scores show higher correlation coefficients with several subscales than the conventional LEAS scores. Together with the results from the comparison to a healthy control group, this strengthens the idea of adding an adequacy rating to the conventional LEAS rating to capture the full construct of EA in patients with schizophrenia and schizoaffective disorders. Results indicate that both, conventional LEAS and adequacy scoring, may relate to structural achievements conceptualized in psychodynamic theory and may be a possible surrogate marker for structural capabilities and change on an intrapersonal level as measured by the OPD-LSIA. However, findings point to the need to differentiate between the ability to represent mental states in an increasingly mature manner, and to draw adequate, context-related conclusions on the respective level of representation.

## Limitations and Future Directions

Conclusion from this study may be limited by methodological weaknesses concerning the adequacy ratings. They were collected after completing the conventional scoring procedure which is based on individual words extracted from the participants’ answers. Whereas rating individual words led to an economic rating procedure that maintained comparability to the conventional LEAS scoring, it might have not led to the best possible operationalization of adequacy in terms of reliability and validity. Specifically, the dispersion of adequacy values within the patient sample was very low and the distribution was highly skewed which pointed toward potential ceiling effects. Future studies should consider collecting adequacy ratings based on full transcripts or audio recordings of participants’ answers such that contextual information can better be incorporated into ratings and a more nuanced evaluation of adequacy can be reached. In addition, adequacy ratings in the present study were provided by an all-female, white rater sample and future work should improve on the diversity of raters.

Although patients and controls were matched for sex, age, and education degree, and interrater reliability between American and German ratings of the conventional LEAS rating of the control sample were excellent, possible differences between an American control sample and a German patient sample due to cross-cultural or language differences cannot be excluded. A comparison especially of the adequacy rating should be done again on a same-language sample to be able to exclude biases due to language differences. This seems to be less problematic with the conventional LEAS rating since objectivity is ensured by the glossary. Moreover, the ways of acquiring the LEAS scores (orally vs. written) were different in the patient and control group. The study of Roberton et al. (2020) did not consider patient groups, which is why we address this problem as a possible limitation. One might argue that the social situation of an interview could influence, e.g., the length and elaborateness of an answer, hence LEAS and adequacy scores. However, the instruction of the interviewers was to only read the instruction and the scenarios aloud without any further interaction. Further, the social situation could increase patients’ answers as well as decrease it, e.g., depending on the respective tendencies of social avoidance or perceiving support and motivation. As we only found a difference between groups in one of the subscales, we consider the likelihood of a systematic influence of the administration mode sufficiently low. Patients and controls differed significantly in educational degree with higher degrees in the control sample. However, it would be expected that this would result in rather higher than lower conventional LEAS scores and adequacy in the control group and would have rather elucidate a difference between patients and controls than confound it. Overall, the level of education was relatively high in both groups. An up-to-date norm sample would also be desirable. However, the sample, of which the control sample examined here was a subsample, has been used in various studies in the past. Isaacowitz et al. (2007) found age differences in recognition of emotion on the basis of the sample and Wright et al. (2018) reported a mediator effect of individual LEAS scores on the relationship between sex and emotion recognition ability.

Another caveat concerns a potential lack of external validity of the LEAS. Lysaker et al. (2010) argued that impairments in metacognitive abilities might become most evident in situations with high emotional arousal and might remain undetected in abstract, experimental tasks. The same could apply to bias tendencies in the context of an adequacy rating and distortions might primarily show up in situations with increased emotional arousal. Future work might resolve this by asking participants to indicate which of the described scenarios they perceive as particularly arousing and by considering this when scoring their responses.

Our patient sample consisted of stabilized or largely remitted patients. Results should therefore be replicated in independent, e.g., transdiagnostic patient samples showing higher levels of positive and negative symptoms, including subgroups with “developmental” or primary negative, as well as acutely delusional or hallucinatory symptoms.

## Data Availability Statement

The raw data supporting the conclusions of this article will be made available by the authors, without undue reservation.

## Ethics Statement

The studies involving human participants were reviewed and approved by Ethics committee of the Charité Universitätsmedizin Berlin. The patients/participants provided their written informed consent to participate in this study.

## Author Contributions

EM wrote the manuscript and performed the statistical analysis. MB organized the database and performed parts of the statistical analysis. RR assisted the statistical questions. All authors contributed to manuscript revision, read, and approved the submitted version.

## Conflict of Interest

The authors declare that the research was conducted in the absence of any commercial or financial relationships that could be construed as a potential conflict of interest.

## Publisher’s Note

All claims expressed in this article are solely those of the authors and do not necessarily represent those of their affiliated organizations, or those of the publisher, the editors and the reviewers. Any product that may be evaluated in this article, or claim that may be made by its manufacturer, is not guaranteed or endorsed by the publisher.

## Abbreviations

LSIA-OPD: Levels of structural integrations axis of the Operationalized Psychodynamic Diagnostics/OPD-2
MAS-A-G: German version of the Metacognition Assessment Scale
MZQ: Mentalization Questionnaire
WST: Wortschatztest (vocabulary test).
