# Supplementary material for: Measuring Emotional Awareness in Patients With Schizophrenia and Schizoaffective Disorders
Source: Front Psychol. 2021 Nov 11;12:725787. doi: 10.3389/fpsyg.2021.725787 (PMC8632138; doi:10.3389/fpsyg.2021.725787)
Supplement: Supplementary file 1 [file Data_Sheet_1.docx]

**Appendix 1** Effective sample sizes due to missingness and exclusions by variable.

| Variable | Patients (initial *N* = 130) | | |  | Controls (initial *N* = 129) | | |
| --- | --- | --- | --- | --- | --- | --- | --- |
|  | Effective *n* | Missings^a^ | Outlier Exclusions^b^ |  | Effective *n* | Missings^a^ | Outlier Exclusions^b^ |
| LEAS total | 123 | 3 | 4 |  | 124 | 0 | 5 |
| LEAS self | 123 | 3 | 4 |  | 124 | 0 | 5 |
| LEAS other | 123 | 3 | 4 |  | 124 | 0 | 5 |
| LEAS total adequacy | 123 | 3 | 4 |  | 122 | 0 | 7 |
| LEAS self adequacy | 123 | 3 | 4 |  | 122 | 0 | 7 |
| LEAS other adequacy | 123 | 3 | 4 |  | 122 | 0 | 7 |
| PANNS | 123 | 0 | 7 |  |  |  |  |
| PANNS adequacy | 123 | 0 | 7 |  |  |  |  |
| MZQ | 97 | 34 | 4 |  |  |  |  |
| MZQ adequacy | 97 | 34 | 4 |  |  |  |  |
| MAS-A | 123 | 0 | 7 |  |  |  |  |
| MAS-A adequacy | 123 | 0 | 7 |  |  |  |  |
| OPD-2 | 123 | 0 | 7 |  |  |  |  |
| OPD-2 adequacy | 123 | 0 | 7 |  |  |  |  |
| AVLT | 120 | 6 | 4 |  |  |  |  |
| AVLT adequacy | 119 | 7 | 4 |  |  |  |  |
| WST | 122 | 4 | 4 |  |  |  |  |
| WST adequacy | 122 | 4 | 4 |  |  |  |  |

*^a^ Missing values occurred either because participants did not send back their questionnaires (MZQ) or because the task was not performed (AVLT and WST, LEAS). ^b^ Outliers were defined as values deviating by more than two standard deviations from the sample mean.*

**Appendix 2** Interrater reliabilities for adequacy for LEAS *Self* and *Other* responses separated by version A and B of *k* = 5 raters for all 10 scenarios.

|  | ICC [95%CI] | | | |
| --- | --- | --- | --- | --- |
|  | Self | | Other | |
| Scenarios | Version A | Version B | Version A | Version B |
| scenario 1 | 0.71 [0.58; 0.81] | 0.86 [0.78; 0.91] | 0.79 [0.69; 0.87] | 0.94 [0.91; 0.97] |
| scenario 2 | 0.70 [0.56; 0.80] | 0.68 [0.53; 0.79] | 0.73 [0.62; 0.82] | 0.82 [0.74; 0.89] |
| scenario 3 | 0.82 [0.74; 0.89] | 0.78 [0.68; 0.86] | 0.93 [0.89; 0.95] | 0.89 [0.85; 0.93] |
| scenario 4 | 0.63 [0.47; 0.75] | 0.69 [0.52; 0.81] | 0.70 [0.57; 0.81] | 0.91 [0.85; 0.95] |
| scenario 5 | 0.78 [0.67; 0.85] | 0.85 [0.77; 0.91] | 0.82 [0.74; 0.88] | 0.86 [0.80; 0.91] |
| scenario 6 | 0.81 [0.73; 0.88] | 0.64 [0.47; 0.77] | 0.89 [0.84; 0.92] | 0.82 [0.73; 0.89] |
| scenario 7 | 0.65 [0.50; 0.77] | 0.52 [0.31; 0.68] | 0.88 [0.83; 0.92] | 0.87 [0.81; 0.92] |
| scenario 8 | 0.72 [0.60; 0.81] | 0.74[0.62; 0.82] | 0.87 [0.82; 0.91] | 0.83 [0.74; 0.88] |
| scenario 9 | 0.72 [0.58; 0.82] | 0.49 [0.29; 0.65] | 0.86 [0.79; 0.91] | 0.77 [0.66; 0.85] |
| scenario 10 | 0.79 [0.68; 0.82] | 0.78 [0.68; 0.86] | 0.80 [0.70; 0.87] | 0.80 [0.69; 0.88] |

*ICC = Intraclass Correlation coefficients based on a mean-rating (k = 5), absolute-agreement, 2-way mixed-effects model; [95% CI] = 95% Confidence Interval [lower bound; upper bound].*
